# Shifting the paradigm in *Dirofilaria immitis* prevention: blocking transmission from mosquitoes to dogs using repellents/insecticides and macrocyclic lactone prevention as part of a multimodal approach

**DOI:** 10.1186/s13071-017-2438-4

**Published:** 2017-11-09

**Authors:** John W. McCall, Marie Varloud, Elizabeth Hodgkins, Abdelmoneim Mansour, Utami DiCosty, Scott McCall, James Carmichael, Ben Carson, Justin Carter

**Affiliations:** 1 TRS Labs, Inc., 215 Paradise Boulevard, Athens, GA 30607 USA; 2 Ceva Santé Animale, Libourne, France; 3 Ceva Animal Health, Lenexa, Kansas USA

**Keywords:** Prevention strategy, *Dirofilaria immitis*, Infection, Lethal repellency, Vector, Mosquitoes

## Abstract

**Background:**

This study assessed the influence of a topical ectoparasiticide (dinotefuran-permethrin-pyriproxyfen, DPP, Vectra® 3D, Ceva Animal Health) combined with a macrocyclic lactone (milbemycin oxime, MBO, Interceptor®, Virbac) on transmission of heartworm L3 from mosquitoes to dogs and subsequent development of worms in treated dogs exposed to infected mosquitoes.

**Methods:**

Thirty-two beagle dogs were allocated to four groups of eight: Group 1, untreated controls; Group 2, treated topically with DPP on Day 0; Group 3, treated orally with MBO on Day 51; and Group 4, treated with DPP on Day 0 and MBO on Day 51. Dogs were exposed under sedation for 1 h to *Dirofilaria immitis* (JYD-34)-infected *Aedes aegypti* on Days 21 and 28. At the end of each exposure, mosquitoes were classified as live, moribund, or dead and engorged or non-engorged. Live or moribund mosquitoes were incubated for daily survival assessment for 3 days. Mosquitoes were dissected before and after exposure to estimate the number of L3 transmitted to each dog. Dogs were necropsied 148 to 149 days postinfection.

**Results:**

A total of 418 mosquitoes fed on the 16 dogs in Groups 1 and 3, while only 6 fed on the 16 DPP-treated dogs in Groups 2 and 4. Mosquito anti-feeding (repellency) effect in Groups 2 and 4 was 98.1 and 99.1%, respectively. The estimated numbers of L3 transmitted to controls, DPP-treated, MBO-treated and DPP + MBO-treated dogs were 76, 2, 78, and 1, respectively. No heartworms were detected in any of the DPP + MBO-treated dogs (100% efficacy), while 8 out of 8 were infected in the control group (range, 21–66 worms per dog), 8 out of 8 were infected in the MBO-treated group (58% efficacy), and 3 out of 8 were infected in the DPP-treated group (96% efficacy).

**Conclusions:**

DPP repelled and killed most mosquitoes that were capable of transmitting heartworm L3 to dogs. The “Double Defense” protocol of DPP + MBO had better efficacy for protecting dogs against heartworm transmission and infection than MBO alone. This added DPP benefit is more pronounced when macrocyclic lactone-resistant strains of heartworms are involved or lack of compliance in macrocyclic lactone administration is known or suspected.

## Background

Heartworm (*Dirofilaria immitis*) is generally considered the most important vector-borne disease of dogs in the United States and many other parts of the world [1, 2]. Cats, ferrets, wild canids and felids, numerous other animals and even humans are also susceptible to infection; and some of these animals add substantially to the pool of microfilaremic reservoirs of infection [2, 3] in warm, humid regions throughout the world. The disease can be life-threatening to our infected pets, and these animals face risky and expensive treatment to clear the infection or manage multi-organ pathology in infected animals. The disease can be prevented, and it is far better to prevent infection than to treat the animal that is infected with adult heartworms and microfilariae.

Macrocyclic lactone (ML) preventive drugs have been widely used for the past three decades [2]. These preventive drugs are safe, highly effective, easy to administer and relatively inexpensive, particularly when compared with treatment. Even with these excellent products readily available, however, the prevalence of heartworm disease has not declined; and one report suggests that the number of positive cases compared with the total number of dogs tested in the United States increased by 15.3% from 2013 to 2016 (www.capcvet.org; accessed April 26, 2017).This is generally attributed to owner and clinic lack of compliance, as less than 40% of dogs leave veterinary hospitals with preventive medication, and inconsistent dosing by owners further adds to prevention failures [4].

It is now well-documented that resistance to all ML has occurred, particularly in the Mississippi Delta area of the United States; however, the extent and degree of spreading of resistant populations of worms is still not known [5, 6]. In Australia, recent evidence indicates a suspicion of heartworm resistance [7]. Once a resistant gene(s) is established in a population of heartworms, ML drug pressure further selects for resistant worms, ML will become less effective over time; and mosquitoes, if not repelled or killed, will spread these resistant worms to animals in other geographical areas.

Apparently many species of mosquitoes can serve as vectors of the heartworm parasite, and at least one species of vector mosquitoes in any suitable climate can live in even a small amount of water, either polluted or clean. More than 70 different species of mosquitoes have been shown to allow development of microfilariae to infective third-stage larvae (L3) in the laboratory [3], and the limited number of field studies to date have detected more than 20 different species of mosquitoes with L3 [8–10]. The high susceptibility of numerous animals, especially dogs and wild canids, along with the ubiquitous presence of susceptible mosquitoes and inadequate heartworm preventive measures and vector control, ensures a high prevalence of heartworm disease in pets in most warm, humid climates of the world.

Arthropod-targeted disease preventive strategies in a multimodal approach to controlling several vector-borne human diseases have been used with much success for several decades. For example, the use of permethrin-impregnated bed nets and clothing, repellents and various vector control measures has been invaluable in blocking the transmission of malaria, lymphatic filariasis, onchocerciasis and some viral diseases, as well as preventing vector biting (Robert Wirtz, personal oral communication, March 2016). For the past three decades, veterinarians have used with much success the unimodal approach of killing heartworm larvae by monthly, semiannual or annual (Australia) administration of ML preventives after the animal has become infected, but mosquito control has received little attention. The American Heartworm Society canine guidelines [1] recommend “…environmental control measures, including treatment of standing water sources with insect growth regulators (IGR) combined with mosquito adulticidal measures (sprays, CO_2_ traps, etc). In addition to mosquito control, keeping pets inside during peak mosquito hours and/or the use of mosquito repellents on pets may also reduce the risk of infection.” Despite this recommendation, the veterinary profession has never focused on vector control as an integral part of a multimodal approach to blocking heartworm transmission. This is probably due mainly to the dearth of research data on the subject.

Several studies have reported varying levels of success with the use of topically applied products for repelling and/or killing several different species of mosquitoes [11–13]. One study assessed the infection of dogs and cats by West Nile virus– infected mosquitoes [14], but only one of these studies included mosquitoes or animals infected with *D. immitis* [15]. Encouraged by the high level of effectiveness of one of these products (Vectra® 3D, dinotefuran-permethrin-pyriproxyfen, DPP; Ceva Animal Health) against *Aedes aegypti* [16] and *Culex pipiens* [17], we investigated the role of DPP in blocking heartworm parasite transmission in dogs in two studies. The first of these two studies was an exploratory study with microfilaremic dogs [18]. In that study we confirmed that DPP was more than 95% effective in repelling (anti-feeding) and killing mosquitoes for 1 month. In the same study, we demonstrated that DPP was completely effective in killing the few mosquitoes that fed on the treated dogs before they lived long enough for the microfilariae to develop to L3 and, consequently, was completely effective in blocking the transmission of L3 to other animals [18].

The results of the second study are reported herein. The primary objective of this study was to evaluate the effectiveness of milbemycin oxime (MBO) when combined with DPP against experimental infection of dogs exposed to *D. immitis–*infected mosquitoes.

## Methods

This clinical efficacy study was GCP, negative-controlled, single-site and blinded. The products were administered to animals by individuals who were not involved in performing the posttreatment assessments and observations. All personnel making observations, performing tests and procedures, and collecting data were blinded in regard to which were treated and control animals. Groups were color-coded for identification by laboratory personnel throughout the study.

### Study design and schedule

The 32 dogs were ranked by descending body weight (BW) within gender and randomly allocated to four groups of eight dogs each, with equal numbers of male and female dogs in each group. After randomization to treatment blocks, the dogs were further randomly allocated to one of two replicates (A and B), each consisting of 16 dogs in 4 subgroups of 4 dogs each, with equal numbers of male and female dogs (Table 1). The two replicates were run 1 day apart. The dogs in Group 1 served as the untreated controls; those in Group 2 were treated topically with DPP on Day 0; those in Group 3 were treated orally with MBO on Day 51; and those in Group 4 were treated topically with DPP on Day 0 and orally with MBO on Day 51 (Table 2). All dogs were infected on Days 21 and 28 by exposure to infected mosquitoes and necropsy was conducted on Day 176–177 (ie, 148–149 days after the second infection).

**Table 1:** Study design

Replicate	Group 1 control	Group 2 DPP^a^	Group 3 MBO^b^	Group 4 DPP + MBO
A	*N* = 4	N = 4	N = 4	N = 4
B	N = 4	N = 4	N = 4	N = 4
Total	*N* = 8	N = 8	N = 8	N = 8

^a^ *DPP* dinotefuran + permethrin + pyriproxyfen (Vectra® 3D)

^b^ *MBO* milbemycin oxime (Interceptor®)

**Table 2:** Study schedule

Study step	Study day			
DPP^a^ administration in Groups 2 and 4	0			
MBO^b^ administration in Groups 3 and 4	51			
Body weight for randomization	–1			
Microfilaremia + antigen test	–5		141	
Exposure to infected mosquitoes	21	28		
L3 counts in mosquitoes	21	28		
Counts of worms in dogs (necropsy)				176^c^-177

^a^ *DPP* dinotefuran permethrin pyriproxyfen (Vectra® 3D)

^b^ *MBO* milbemycin oxime (Interceptor®)

^c^Replicate A dogs were necropsied on Study Day 177 and replicate B dogs were necropsied on Study Day 176

### Animals

A total of 32 purpose-bred beagles, 16 males and 16 females, 4.8 to 6.7 months of age and weighing 7.5 to 13.7 lb, were involved in this study. Prior to the start of the study, all dogs were bathed with a noninsecticidal grooming shampoo. On Day −5 and again on Day 141 (120 days after the first infection), all dogs were negative on the modified Knott’s test [1] and antigen test (DiroCHEK® Canine Heartworm Antigen Test Kit, Synbiotics). The dogs were housed individually in mosquito-proof indoor pens (5 × 4 ft) in a purpose-built building, with controlled temperature and ventilation systems. The dogs were fed at least once daily an appropriate quantity of commercially available maintenance diet, and water was supplied ad libitum. The animals were maintained with due regard for their welfare and in accordance with applicable laws, regulations and guidelines. The protocol was approved by an ethics committee (TRS Labs’ Institutional Animal Care and Use Committee) prior to initiation of the study.

### Parasite and vector

The JYD-34 isolate of *D. immitis* was used. This isolate is known to have varying degrees of resistance to all of the four ML used for heartworm prevention in dogs [19] (JWM, unpublished data, August 2013). The blackeyed Liverpool strain of *A. aegypti* mosquitoes was used as the vector. Female mosquitoes were raised in one-gallon oyster cartons and infected on microfilaremic blood 16 days prior to dog exposure. The dead mosquitoes were removed daily from each carton and the mosquito count was updated. The targeted number of mosquitoes to be released per dog was matched by prior removal of extra mosquitoes from the boxes.

### Treatments

The dogs in Groups 2 and 4 were treated on Day 0 with DPP (topical solution of dinotefuran 4.95% *w*/w, pyriproxyfen 0.44% w/w and permethrin 36.08% w/w; Vectra^®^ 3D) at an average rate of 0.35 ± 0.06 mL/kg BW (Table 3). The product was applied topically according to the label, as a line-on from the base of the tail to the shoulders. The dogs in Groups 3 and 4 were treated orally with MBO (tablet of milbemycin oxime, Interceptor®) 30 days after the first infestation (ie, on Day 51). According to the instructions on the label and to the BW measured on Day 47 ± 2, an average dose of 0.92 ± 0.15 mg/kg BW of milbemycin oxime was delivered to the dogs (Table 3). The control dogs (Group 1) were not treated. For each treatment, the dogs were under observation every hour (±15 min) for the first 4 h after the last animal was treated.

**Table 3:** Dogs and treatment

Group	Treatment	Body Weight (lb, Day −1)	DPP^a^ Volume (mL, Day 0)	MBO^b^ (mg, Day 51)
1	Control	10.1 ± 2.0	–	–
2	DPP^a^	10.2 ± 1.8	1.6	–
3	MBO^b^	10.3 ± 1.8	–	5.75
4	DPP + MBO	10.3 ± 1.7	1.6	5.75

^a^ *DPP* dinotefuran + permethrin + pyriproxyfen (Vectra® 3D)

^b^ *MBO* milbemycin oxime (Interceptor®)

### Exposure of dogs to infected mosquitoes

**Fig. 1:**
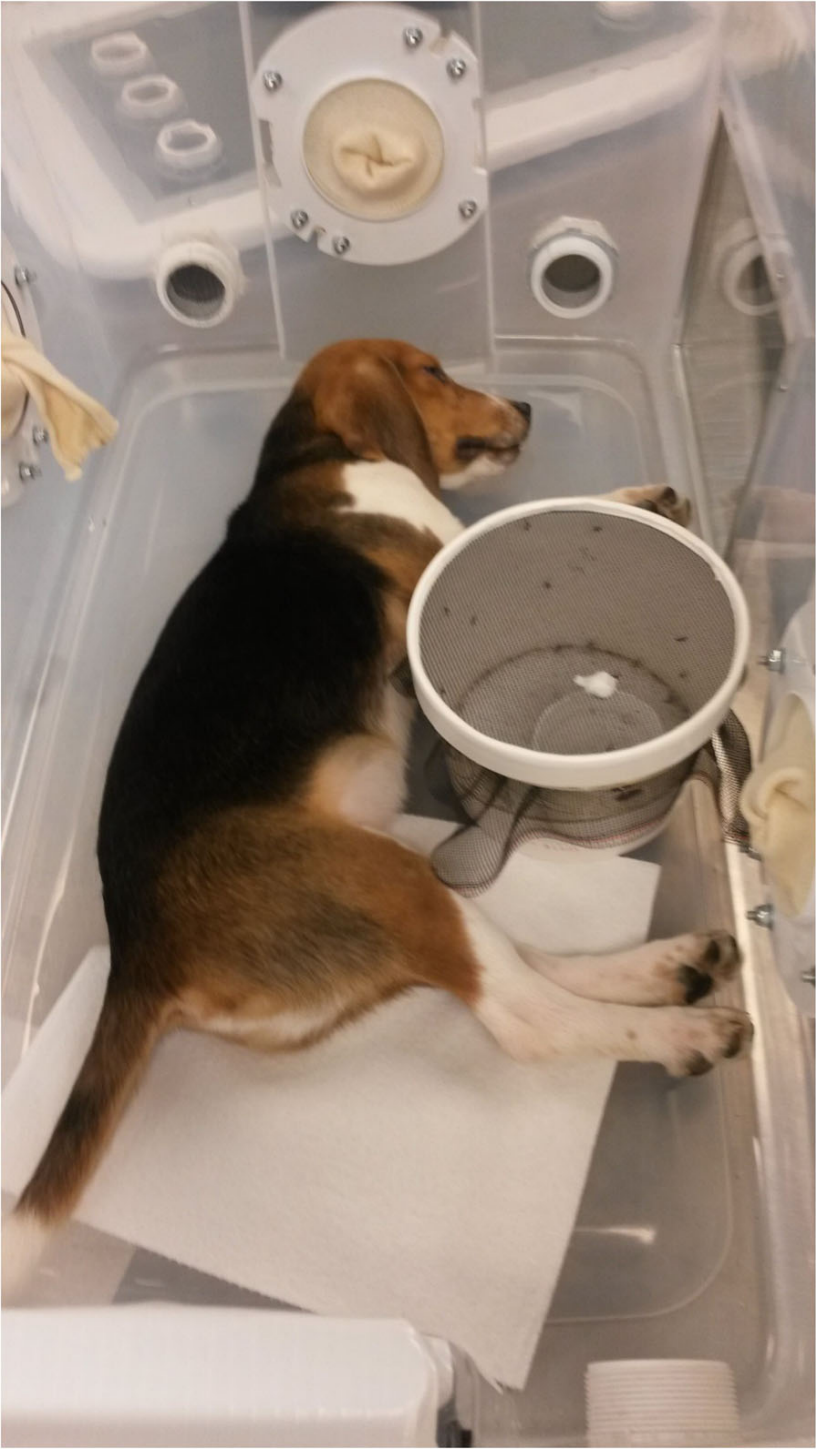
Exposure of a dog to mosquitoes in a mosquito-proof container prior to the release of nonblood-fed *D. immitis* (JYD-34 strain)-infected female mosquitoes (*Aedes aegypti*) from the carton

All dogs were exposed to mosquitoes infected with *D. immitis* L3 on Days 21 and 28. Prior to each exposure, each dog was sedated by IM injection of dexmedetomidine 0.02 mg/kg BW (Dexdomitor®, Orion, Espoo, Finland) and butorphanol 0.2 mg/kg BW (Torbugesic®, Zoetis). Each dog was placed in a dedicated container (73.7 cm long × 40.6 cm wide × 33 cm high), and the lid was replaced, making the container mosquito-proof (Fig. 1). The infected mosquitoes were released into the container and the dog was exposed to the infected mosquitoes for 60 (±10) minutes. The procedure was conducted during the day and under artificial light.

Immediately after exposure, mosquitoes were aspirated from the container and categorized as live, moribund or dead, and fed or unfed. A mosquito was considered live when it exhibited normal behavior and was capable of flying. Moribund mosquitoes were unable to move normally and clearly exhibited neurological disruption. Mosquitoes with red and enlarged abdomens were considered engorged. Dead mosquitoes were preserved in ethanol (70%) and stored at −20 °C. Live and moribund mosquitoes from each container were placed in an insectary (28 °C, 80% RH), and mortality was assessed daily for 3 days.

### Estimation of number of L3 transmitted to each dog

Prior to each exposure, for each replicate of 16 dogs, approximately 48 mosquitoes (~3 from each carton) were dissected individually to determine the average number of *D. immitis* L3 per mosquito. Based on this calculation, a sufficient number of infected mosquitoes to transmit ~25 L3/dog/exposure for a total of ~40–60 L3/dog/2 exposures was made available in each carton. During each exposure period, up to three blood-engorged mosquitoes were removed from each container and dissected individually to determine the average number of L3 remaining in the mosquitoes after feeding. The estimated number of L3 transmitted to each of the 16 dogs in each replicate and for each exposure was calculated by subtracting the average number of L3 remaining in each mosquito after feeding from the average number of L3 in each mosquito prior to feeding, and then multiplying this number by the number of mosquitoes that fed on that dog.

### Worm counts

On Days 176–177 all dogs were humanely euthanized and necropsied for recovery and enumeration of adult worms in the pleural and peritoneal cavities and the heart and associated pulmonary arteries. At necropsy, all worms were recovered and enumerated by gender. Mortality was assessed by observing the motility of each worm in saline. Worms that were immotile in warm saline were considered dead.

### Data analysis

#### Anti-feeding effect or repellency

For each day of exposure (Days 21 and 28), the anti-feeding effect was calculated:

$$ \mathrm{Anti}\hbox{-} \mathrm{feeding}\  \mathrm{effect}\ \left(\%\right)=100\times \frac{\left(\mathrm{Cf}\hbox{--} \mathrm{Tf}\right)}{Cf} $$

Where **Cf** was the geometric mean of fed female mosquitoes (live fed + dead fed) in Groups 1 and 3 (no-DPP), and **Tf** was the geometric mean of the fed female mosquitoes in Groups 2 and 4 (DPP treated).

#### Knock-down effect

For each day of exposure (Days 21 and 28), the knock-down effect was calculated:

$$ \mathrm{Knock}\hbox{-} \mathrm{down}\  \mathrm{effect}\ \left(\%\right)=100\times \frac{\mathrm{Cl}\hbox{--} \mathrm{Tl}}{\mathrm{CI}} $$

Where **Cl** was the geometric mean of live female mosquitoes (live engorged + live unengorged) in Groups 1 and 3 (no DPP), and **Tl** was the geometric mean of the live female mosquitoes in Groups 2 and 4 (DPP treated).

The knock-down effect was calculated based on the data collected at the end of the exposure (1 h).

#### Insecticidal effect

For each day of exposure (Days 21 and 28), the mortality effect was calculated:

$$ \mathrm{Mortality}\  \mathrm{effect}\ \left(\%\right)=100\times \frac{\left(\mathrm{Clm}\hbox{--} \mathrm{Tlm}\right)}{\mathrm{Clm}} $$

Where **Clm** was the geometric mean of live + moribund female mosquitoes (live engorged + live unengorged + moribund engorged + moribund unengorged) in Groups 1 and 3 (no-DPP), and **Tlm** was the geometric mean of the live + moribund female mosquitoes in Groups 2 and 4 (DPP treated). The mortality effect was calculated daily for 3 days of the postexposure incubation.

#### *Theoretical exposure of dogs to* D. Immitis *L3*

The estimated number of L3 deposited on each dog was calculated by taking into account the number of fed mosquitoes and the counts of L3 in mosquitoes prior to and after blood-feeding on dogs.

$$ \mathrm{L}3\ \mathrm{transmitted}\ \mathrm{per}\ \mathrm{dog}=\mathrm{NFed}\times \left(\mathrm{L}3\ \mathrm{before}\hbox{--} \mathrm{L}3\ \mathrm{after}\right) $$

Where **NFed** was the cumulated number of mosquitoes fed on dogs for each dog, **L3 before** was the average number of L3 found in mosquitoes upon dissection before exposure of the dogs and **L3 after** was the average number of L3 found in fed mosquitoes upon dissection after exposure of the dogs.

#### Heartworm preventive efficacy

The heartworm preventive efficacy was calculated using the worm counts found at necropsy (Days 176–177) in the circulatory system of the dogs.

$$ \mathrm{Preventive}\  \mathrm{efficacy}\ \left(\%\right)=100\times \frac{\left(\mathrm{Cw}\hbox{--} \mathrm{Tw}\right)}{\mathrm{Cw}} $$

Where **Cw** was the arithmetic mean number of worms found at necropsy of dogs from Group 1 (Control), and **Tw** was the arithmetic mean number of worms found at necropsy of dogs from each of the treated groups (Group 2 or Group 3 or Group 4).

#### Statistics

For the primary endpoint, a Poisson model (GENMOD proc., SAS) was used for comparison of the L3 estimated number and of the heartworm counts between treatments: (1) DPP (Group 2) vs DPP + MBO (Group 4) and (2) DPP (Group 2) vs untreated control (Group 1). The “fed” status of mosquitoes was considered a covariable. For the secondary endpoint, a Friedman ANOVA model (rank transformation for normalization) was used for comparison of the number of fed or live mosquitoes between DPP-treated groups (Groups 2 and 4) and non-DPP-treated groups (Groups 1 and 3).

### Guidelines

This study was carried out in compliance with Good Clinical Practice requirements (VICH GL 9, 2001), with FDA Guidance No. 111 and with US EPA Product Performance Test Guidelines OPPTS 810.3300: Treatments to Control Pests of Humans and Pets.

## Results

No adverse effects due to any of the treatment applications were observed in any dogs during the study.

### Anti-feeding efficacy

In the no-DPP Groups (1 and 3, *n* = 16), mosquito blood-feeding occurred in all the dogs. The number of fed mosquitoes per dog ranged from 7 to 24 on Day 21 and from 6 to 17 on Day 28 (Table 4). The feeding rate in the no-DPP groups was 43% on Day 21 and 50% on Day 28. In the DPP Groups (2 and 4, n = 16), the number of fed mosquitoes per dog ranged from 0 (*n* = 15) to 1 (n = 1) on Day 21 and from 0 (*n* = 13) to 3 on Day 28. On Day 28, mosquito blood-feeding was detected in 3 out of 16 dogs from the DPP-treated groups. On both days of mosquito exposure, the number of fed mosquitoes was higher (*P* < 0.0001) in the no-DPP groups than in the DPP groups. The anti-feeding efficacy of DPP assessed 3 and 4 weeks after treatment was 99.7% and 98.4%, respectively. The overall anti-feeding efficacy against the 867 mosquitoes released on the DPP-treated dogs on Days 21and 28 was 99.0%.

**Table 4:** Geometric mean number of blood-fed and live mosquitoes per dog and immediate anti-feeding and knock-down efficacy (%) of DPP* after 1 h exposure (%) on Days 21 and 28 after administration in Group 2 and 4 (*n* = 16) dogs

	Groups 1 and 3 (untreated^a^)		Groups 2 and 4 (DPP^b^)		Efficacy (%)	
	Mean/Total (range)		Mean/Total (range)			
Day	Fed	Live	Fed	Live	Anti-feeding	Knock-down
21	13.1/30.4 (7–24)	28.3/30.4 (24–40)	0.0/30.7 (0–1)	2.3/30.7 (0–11)	99.7	91.8
28	11.7/23.4 (6–17)	21.5/23.4 (17–33)	0.2/23.5 (0–3)	1.8/23.5 (0–10)	98.4	91.9
Overall					99.0	91.8

^a^Untreated at the time of the mosquito exposure

^b^DPP: dinotefuran + permethrin + pyriproxyfen (Vectra® 3D) applied on Day 0

### Knock-down efficacy

In the no-DPP groups (1 and 3, *n* = 16), the mosquitoes did not exhibit signs of knock-down or death. The average proportion of live mosquitoes was 93.2% and 91.7% after exposure on Days 21 and 28, respectively (Table 4). In the DPP groups (2 and 4, *n* = 16), most of the mosquitoes showed impaired coordination and signs of death. The average proportion of live mosquitoes was 7.4% and 7.7% after exposure on Days 21 and 28, respectively (data not shown). On both days of mosquito exposure, the number of live mosquitoes was higher (*P* < 0.0001) in the no-DPP groups than in the DPP groups. The knock-down efficacy of DPP assessed 3 and 4 weeks after treatment was 91.8% and 91.9%, respectively. The overall knock-down efficacy against the 867 mosquitoes released on the DPP-treated dogs on Days 21 and 28 was 91.8%.

### Insecticidal efficacy

The survival of the total cumulated 1600 mosquitoes found live or moribund at the end of the 1-h exposure to dogs was assessed daily over 3 days after each exposure day (Table 5). In the no-DPP groups (1 and 3, n = 16), the average survival of mosquitoes per dog was 73.5% (20.0 out of 27.2 incubated) and 80.7% (17.6 out of 21.8 incubated) 24 h after exposure on Days 21 and 28, respectively. The survival of the mosquitoes from the no-DPP groups decreased during the incubation and was recorded as 61.8% and 69.7% when assessed at 72 h after exposure on Days 21 and 28, respectively (data not shown). In the DPP groups (2 and 4, *n* = 16), the average survival of mosquitoes per dog was 12.7% (3.6 out of 28.4 incubated) and 15.5% (3.5 out of 22.6 incubated) 24 h after exposure on Days 21 and 28, respectively. After both mosquito exposure days, the number of mosquitoes dying during incubation was higher (*P* < 0.0001) in the DPP groups than in the no-DPP groups. After 24 h of incubation of live or moribund mosquitoes collected after dog exposure, the insecticidal efficacy of DPP was 81.8% and 80.4%, 3 and 4 weeks after DPP administration, respectively. The overall insecticidal efficacy of DPP against infected *A. aegypti* mosquitoes assessed after 24 h of incubation postexposure was 81.1%.

**Table 5:** Geometric mean number of live or moribund mosquitoes per dog and insecticidal efficacy (%) of DPP after 24, 48 and 72 h of incubation after exposure on Days 21 and 28

Exposure Day	Groups 1 and 3 (untreated^a^) Incubation Time (h)			Groups 2 and 4 (DPP^b^) Incubation Time (h)		
	24	48	72	24	48	72
21	20.0/27.2^c^	18.3^d^	16.8^d^	3.6/28.4 81.8%	3.5 80.7%	3.5 78.9%
28	17.6/21.8	16.5	15.2	3.5/22.6 80.4%	3.2 80.3%	3.2 78.6%
Overall efficacy				81.1%	80.5%	78.7%

^a^Untreated at the time of the mosquito exposure

^b^ *DPP* dinotefuran + permethrin + pyriproxyfen (Vectra® 3D) applied on Day 0

^c^Mean number surviving/total recovered

^d^Mean number surviving

### Theoretical L3 transmission

Estimated numbers of *D. immitis* L3 deposited per dog were calculated based on the number of fed mosquitoes and the number of L3 detected in mosquitoes before and after blood-feeding (Table 6). Whatever the group, the average number of L_3_ per mosquito before exposure and blood-feeding (*n* = 192) ranged from 3.2 (0–13) to 4.6 (0–21) on Days 21 and 28, respectively. After blood-feeding, the average number of L3 per mosquito (*n* = 79) was 0.8 (0–8) on both days of exposure (data not shown). In the no-DPP groups (1 and 3, *n* = 16), the estimated number of L3 deposited per dog was 32.2 and 45.3 on Days 21 and 28 (data not shown), respectively. In the DPP groups (2 and 4, n = 16), the estimated number of L3 deposited per dog was 0.1 and 1.1 on Days 21 and 28, respectively (data not shown). The total expected number of L3 deposited per dog after the two exposures to infected mosquitoes was higher (*P* < 0.0001) in Groups 1 and 3 (76 and 78, respectively) than in Groups 2 and 4 (2 and 1, respectively).

**Table 6:** Average number of *Dirofilaria immitis* (JYD-34 strain) infected mosquitoes (*Aedes aegypti*), average L3 load of mosquitoes used to infect dogs and average L3 potentially deposited on dogs

Groups	1	2	3	4
Day 21				
Mosquitoes released (n/dog)	29.4 ± 3.5	32.1 ± 6.4	31.4 ± 5.3	29.3 ± 3.0
L3 before feeding/mosquito (n dissected mosquitoes)	3.2 (24)	3.2 (28)	3.5 (24)	2.9 (20)
L3 after feeding/fed mosquito (n dissected fed mosquitoes)	1.3 (12)	1.0 (1)	0.3 (12)	N/A* N/A*
Fed mosquitoes/dog	15 ± 5.8	0.1 ± 0.4	12.5 ± 5.7	0 ± 0
L3 deposited/dog	33.4 ± 8.5	0.2 ± 0.6	31.0 ± 6.2	0 ± 0
Day 28				
Mosquitoes released (n/dog)	21.5 ± 2.7	24.1 ± 4.1	25.4 ± 8.3	22.9 ± 2.9
L3 before feeding/mosquito (n dissected mosquitoes)	5.4 (24)	4.7 (27)	3.7 (24)	4.6 (21)
L3 after feeding/fed mosquito (n dissected fed mosquitoes)	1.0 (24)	0.3 (3)	0.7 (24)	0.3 (3)
Fed mosquitoes/dog	11.5 ± 3.3	0.4 ± 1.1	12.6 ± 2.6	0.3 ± 0.5
L3 deposited/dog	43.3 ± 12.5	1.3 ± 3.6	47.4 ± 9.1	0.9 ± 1.8
Estimated total L3 deposited/dog	76 ± 11	2 ± 3	78 ± 14	1 ± 2

*N/A = No mosquitoes in this group fed on this date, therefore no mosquitoes were dissected

### L3 transmission/development blocking efficacy

**Fig. 2:**
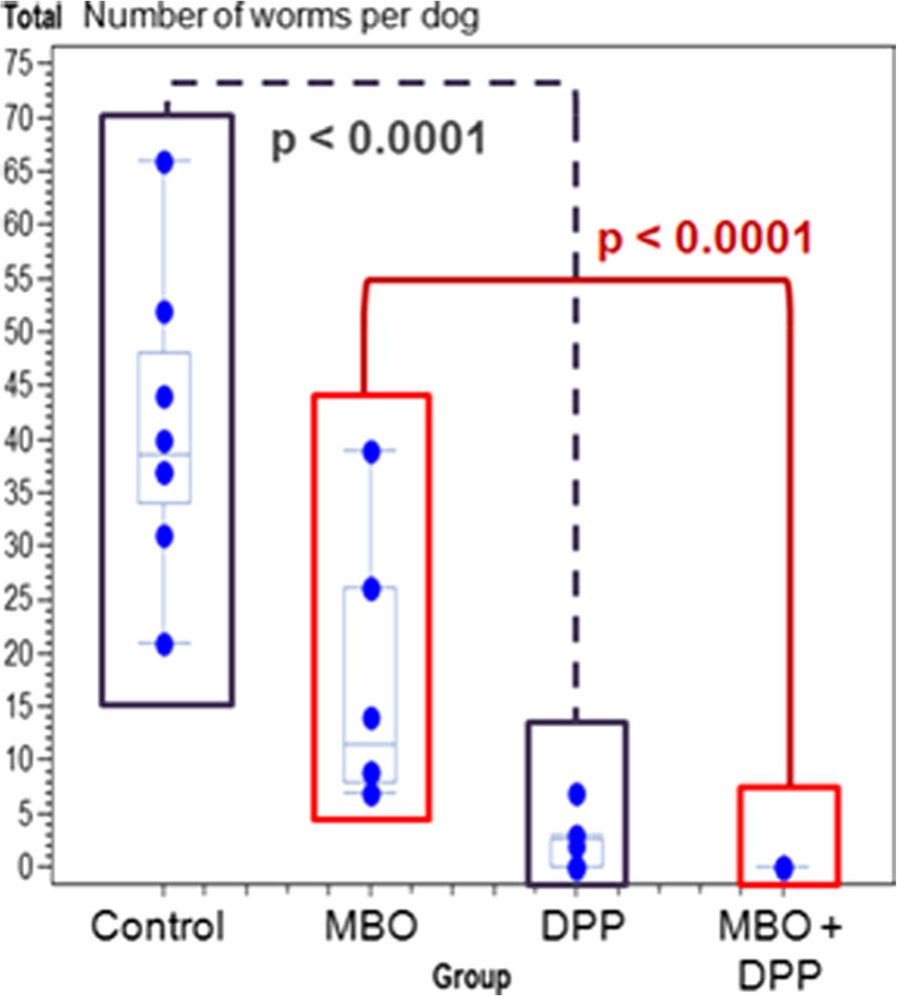
Number of worms at necropsy 148 to 149 days after the last of two weekly exposures to infected mosquitoes (Days 21 and 28) in dogs either untreated, treated topically with DPP^*^(Day 0), treated orally with MBO^†^ (Day 51) or treated with DPP (Day 0) and MBO (Day 51)^‡^. ^*^DPP: dinotefuran + permethrin + pyriproxyfen (Vectra® 3D). ^†^ MBO: milbemycin oxime (Interceptor®) ^‡^See Table 7 for details

At necropsy, no worms were found outside of the circulatory system in any of the dogs, and all of the worms were viable based on their motility and appearance observed in saline after collection. The *D. immitis* worm counts were assessed on each dog on Days 176–177 (Table 7). In Groups 1 and 3, all the dogs were infected with at least 7 worms. In Group 2, 5 dogs were free of worms. None of the Group 4 dogs was infected. In Groups 1, 2, 3 and 4 the average (range) worm burden was 41.0 (21–66), 1.5 (0–7), 17.1 (7–39), and 0.0 (0–0) *D. immitis* per dog, respectively. Two of the dogs from Group 2 with no fed mosquitoes observed on Days 21 and 28 had a few (2 and 3) worms. The worm counts were lower (*p* < 0.0001) in the DPP-treated group (2) as compared to the untreated group (1) and in the DPP + MBO treated group (4) as compared to the MBO-treated group (3) (Fig. 2). The heartworm preventive efficacy was 58.2% for MBO alone, 96.3% for DPP alone and 100% for DPP combined with MBO.

**Table 7:** Worm counts in dogs 148 to 149 days after the last infection by exposure to *Aedes aegypti* mosquitoes previously infected with *Dirofilaria immitis* (JYD-34 strain) 21 and 28 days after DPP administration

Groups	1 Control		2 DPP^a^		3 MBO^b^		4 DPP + MBO	
Worms^c^	Male	Female	Male	Female	Male	Female	Male	Female
Dogs								
1	8	13	3	4	3	4	0	0
2	12	19	1	2	14	12	0	0
3	24	20	0	0	3	4	0	0
4	21	16	0	2	19	20	0	0
5	18	19	0	0	9	5	0	0
6	32	34	0	0	7	2	0	0
7	27	25	0	0	6	3	0	0
8	16	24	0	0	13	13	0	0
Average/dog	19.8	21.3	0.5	1.0	9.3	7.9	0	0
	41.0		1.5		17.1		0	
*P* value	*P* < 0.0001^d^				*P* < 0.0001			
Preventive efficacy (%)	–		96.3		58.2		100	

^a^ *DPP* dinotefuran + permethrin + pyriproxyfen

^b^ *MBO* milbemycin oxime

^c^All worms were live at the time of necropsy and found in the circulatory system

^d^Significant difference between Groups 1 and 2 and between Groups 3 and 4

## Discussion

### Methodological considerations

The model was considered successful since the control group was infected at rates (21–64 worms/dog) close to those usually targeted with other experimental infection techniques, such as the use of subcutaneous injection of L3 (up to 37 worms/dog) [20, 21], or to natural worm burdens where up to 85 worms/dog were found [22]. This model is expected to be close to the natural exposure of dogs by infected mosquitoes, but higher or lower exposure levels can certainly be achieved because animals can be bitten by uninfected mosquitoes, as well as mosquitoes with variable and unpredictable numbers of L3. A study performed in Italy documented the exposure of dogs that could be bitten by about 80 mosquitoes per night and per individual [23]. This number is probably an underestimation of some common situations at certain locations in the United States. In Alaska, more than 1000 mosquito bites per hour were reported over 4 consecutive hours of exposure to natural infestations by eight volunteers [24]. Unlike in humans, for whom a human biting rate is documented for malaria mosquito vectors [25], a dog biting rate is missing for the competent vectors of heartworm.

### Efficacy against mosquitoes (*A. aegypti*)

The efficacy of DPP against mosquitoes was assessed using 32 dogs that were either treated with DPP (*n* = 16, Groups 2 and 4) on Day 0 or untreated with DPP (n = 16, Groups 1 and 3) at the time of mosquito exposures that occurred on Days 21 and 28.

The anti-feeding efficacy against mosquitoes is a consequence of the repellent effect of the product. It is also directly affected by the feeding performance of the mosquitoes on the untreated animals. After release of the infected mosquitoes into the exposure containers with the animals, we observed an overall impaired behavior of the insects as compared to previous observations on uninfected mosquitoes of the same species and strain [18]. Although experimental transmission from dog to dog was recorded [26], to our knowledge, this is the first time that the feeding behavior of free, heartworm-infected *A. aegypti* mosquitoes is documented on dogs. In the present experiment, the mosquitoes were slower to start feeding and did not move as well as usual. The age and health status of mosquitoes is expected to influence their feeding behavior.

In our earlier study [18], the mosquitoes were 4 to 5 days old and not infected, while in the present study, the mosquitoes were 20 to 21 days old and heavily infected with *D. immitis* L3. Feeding behavior is likely to be impaired in older, unhealthy mosquitoes. This disruption of behavior was observed with other filarial-infected *A. aegypti* mosquitoes [27] and can explain the lower feeding rate (42% − 50% in the no-DPP groups) versus 79% to 97% in the previous study with uninfected mosquitoes [18]. An increased duration of exposure (beyond 1 h) was not expected to improve this feeding rate, since the feeding occurred mainly in the first 20 min after release of the mosquitoes.

The insecticidal efficacy of DPP slightly decreased over incubation since the mosquitoes were already over 16 days old, and natural mortality occurred in the DPP-untreated groups as well. This phenomenon greatly contributes to lower the insecticidal efficacy of DPP, especially when compared with assessment performed on younger mosquitoes of the same species [16].

As demonstrated in a side experiment, both effects are only triggered by contact between the treated animal and the insect [28]. There is no evidence of action by vapor-pressure release of any of the DPP active ingredients in the cage, which was under natural ventilation. At 20 °C, the vapor pressure of cis and trans-permethrin is 2.5 and 1.5 μPa [29], respectively; and it only increases to 6.58 μPa above the dog skin temperature at 40 °C [30].

### Efficacy against heartworm (*D. immitis*)

**Fig. 3:**
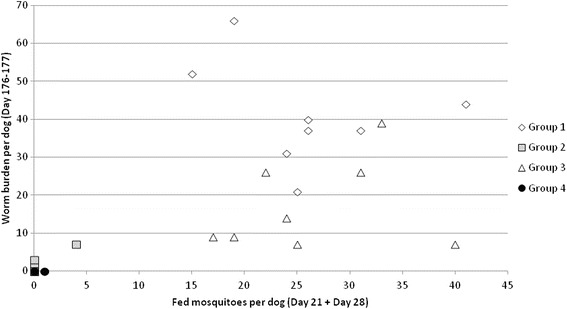
Relationship between the cumulated number of fed infected mosquitoes per dog on Days 21 and 28 and the *Dirofilaria immitis* worm burden in the circulatory system of the untreated (Group 1), DPP*-treated (Group 2), MBO^†^-treated (Group 3) or DPP + MBO-treated (Group 4) dogs on Days 176–177. ^*^DPP: dinotefuran + permethrin + pyriproxyfen (Vectra® 3D). ^†^MBO: milbemycin oxime (Interceptor®)

There was an obvious relationship between the number of fed, infected mosquitoes on dogs and the worm burden determined at necropsy (Fig. 3): a higher number of worms being observed in dogs on which more than 15 mosquitoes were found blood-fed. However, we noticed that worms (*n* = 2 and 3) were found in two of the dogs (Group 2, DPP-treated) for whom no visually fed mosquitoes were recorded at any of the mosquito exposures. This important observation underlines not only that repellent insecticidal products cannot provide 100% protection against mosquito bites, but also that the visual assessment of the engorgement of mosquitoes cannot be considered as a standalone and reliable way to assess the potential heartworm transmission to dogs [18]. In the present experiment, a few, but unknown exact numbers of, mosquitoes were able to transmit heartworm L3 to dogs without visually detectable blood-feeding. Since these dogs treated with DPP were sedated, we assume that their blood-feeding process was disrupted by the treatment and that probing through the skin allowed the deposition of a few heartworm L3.

To our knowledge, the influence of a topical repellent (imidacloprid-permethrin, IP) in the prevention of heartworm transmission has been assessed previously only once [15]. In that experiment, however, the methodology was completely different: there were three dogs per group compared with eight in the present study, the experimental infection of the control untreated dogs (1–4 worms/dog) was lower than the threshold required by FDA (≥5 worms/dog), and the animals were treated with the product 3 or 36 days prior to exposure to *D. immitis*-infected mosquitoes. Whatever the time interval between the administration and the challenge, the anti-feeding efficacy of the repellent tested was markedly lower (70.2% and 72.7%) than DPP (>98%). In two different studies, the anti-feeding efficacy of IP against *A. aegypti* was only 50.4% [12] and 51.5% [11] 28 days after administration. After a topical application of a product containing fipronil and permethrin (FP, not available in the United States), the anti-feeding effect against *A. aegypti* was >96.1%, and the insecticidal efficacy was >80% over a 28-day period [13]. Wide variations in efficacy can also occur between formulations since the anti-feeding effect of a different FP combination tested on dogs against *A. aegypti* was 77% to 91.7% and the insecticidal (killing) efficacy never exceeded 59% during weekly challenges repeated over a 1 month period [31].

In the present study, we selected a challenge based on a well-known resistant heartworm isolate (JYD-34). The efficacy results of MBO against JYD-34 obtained in the present study (58.2%) are within the range of previous efficacy measurements (52.2% after three monthly consecutive administrations and 72.0%, 72.2%, and 76.1% after six, three [19], and two repeated administrations [32], respectively). Until now, all available ML have been shown [19, 32–34] (JWM, unpublished data, March 2013) to fail against this isolate and to experience lack of efficacy reported from the field. Of course, the epidemiology of heartworm resistance in the United States and across worldwide endemic areas is not well known at the present. However, this challenge is representative of other situations in which the ML suffer from insufficient efficacy against heartworm. For example, we know that the risk of efficacy failure will increase with missed chemoprophylactic doses.

This experiment demonstrated the benefit of the addition of insecticidal repellency against the mosquito vectors to larvicidal efficacy against these pathogenic worms. The benefit of the concomitant use of DPP and MBO is an improved reduction of the risk implemented at two levels: transmission to the dog and development of the worms. The two products rely on very different and complementary strategies that can easily be combined in the field for increased protection. Such strategies are already implemented for human health protection. For example, vector control is one of the core measures against dengue [35], malaria and, more recently, Zika virus. In dogs, the combination of a larvicide with an insecticidal-repellent appears to be realistic and achievable by pet owners from an economic point of view since DPP also provides protection against a wide spectrum of ectoparasites that usually infest pets.

## Conclusions

DPP repelled and killed mosquitoes that were capable of transmitting heartworm L3 to dogs. The combination of DPP + MBO had better efficacy for protecting dogs against heartworm transmission and infection than MBO alone. This research supports a “Double Defense” protocol in which DPP can be combined with any heartworm preventive drug. All dogs exposed to heartworm infection should benefit from the mosquito repellency and insecticidal efficacy of DPP added to their heartworm preventive protocol, and this benefit is even more obvious when macrocyclic lactone-resistant strains of heartworm are involved or lack of compliance in the administration of ML preventives is known or suspected.

## Abbreviations

DPP: Dinotefuran-permethrin-pyriproxyfen
IGR: Insect growth regulator
MBO: Milbemycin oxime
MF: Microfilariae
ML: Macrocyclic lactone
